# The Threat from the East and the Tropics

**Published:** 1920-06-26

**Authors:** 


### DISEASE AND CIVILISATION.

### The Threat from the East and the Tropics.

We have just received a copy of one of the most interesting books on overseas disease which have been published within recent times, and which is essentially a collection of papers by Dr. Andrew Balfour, C.B., C.M.G., the Director-in-Chief of the Wellcome Bureau of Scientific Research. The volume combines under the title of "War against Tropical Disease" a series of "seven sanitary sermons addressed to all interested in tropical hygiene and administration," and right valuable sermons they are.

Dr. Balfour outlines a history where practically every medical effort has been doubly and trebly rewarded, and although the training hospitals of England have not perhaps been directly responsible for every one of these successes, yet they have provided the men, they have commenced their training, and it is upon them that the whole fabric of our defences against tropical disease must stand.

### Tropical Sanitation.

To take an example from the conflict with disease, let us select tropical sanitation. As the author well says, it means war, a ceaseless war, and an exhausting war—but, thank God, if properly waged, a successful war—against those conditions which tend to slay the white man and the black. It has been stated that if we could but apply even our present knowledge with full force, all communicable disease could be stamped out in the short space of fifty years. With certain reservation as regards a few particular tropical disorders, we can concur with this optimistic view. What is required not only in the East, but everywhere, is, first, education, and then just that degree of legislation which will crush the active opposition of ignorance—or, for overseas, shall we say fanaticism?—and withal indirectly aid the medical authorities in their work. To realise how much can be done on these principles one has but to read the history of Khartoum. Nowadays, "very different are the conditions from those which rendered Old Khartoum a pest spot and led the wily Dervish to raze it to the ground and establish his capital at Omdurman." Of the new city and its people something may be due to its comparative isolation, something to the fact that it was caught young and early in its new career passed under what might be called a "sanitary tyranny," but, to those who know the habits of these parts, it is remarkable to read that, so far as communicable disease goes, it is doubtful whether a healthier city exists in Africa to-day.

To some of us the idea that the West Indian islands have been in similar urgent need of the "sanitary eye" may come as a mild surprise—but to those who know them and to those who read Dr. Balfour's book this phase of new-world history will be obvious. A point, however, of great interest arises in connection with Barbadoes, which, though in constant shipping contact with malarial-infected countries, does not itself know the disease. There are certainly possible malarial breeding-places, but the porosity of the coral limestone automatically keeps them relatively scarce. Also there are the Barbadoes "millions," a particularly numerous small fresh-water fish, which are numbered among the enemies of malaria. But do the great flocks of migrating birds play havoc with the mosquito larvæ? Dr. Balfour gives us innumerable interesting suggestions, particularly the discovery of a little back-swimmer which feeds voraciously upon culex and stegomyia larvæ, and which, in the complete absence of fish and birds, can keep water absolutely free from malarial mosquitoes. These little beasts have the habit of rising to the surface of the water, drying their wings and flying further afield. Is it possible that their introduction into other lands might help us finally to eradicate malaria?

There is one remarkably interesting chapter in this book, namely that dealing with tropical typhoid and cholera as actually encountered in the hot zones of the world, but of these so much has been written and so well known are the brilliant results of investigational and preventive medicine in the field that we can pass on to a matter experienced by many but understood by but a few: the winged pests of Salonica. A fly—the common fly—has been called by our Canadian friends the typhoid fly, but from the author's own investigation it might just as well be termed the paratyphoid fly, or the dysentery fly, or the cholera fly, or the helminth fly, or possibly even the P.U.O. fly. A thousand interesting relations are being daily recognised. There are the tick-flies which are at least suspected of conveying *Leishmania tropica*, causing the "oriental sore"; their toughness and tenacity are such that the only way to deal with them is to imitate the tactics of Cromwell with Charles I. The yellow-fever mosquito, the various malarial mosquitoes, and a great number of close relatives are gradually meeting with correct classification and study and, thank goodness, with corresponding destructive measures. But even were their obliteration complete, there still remains the vast problem of dealing with their wingless colleagues, and even then we should have touched upon but the fringe of a vast subject. In the civilised temperate zones, such matters are sufficiently important and how vital their significance in the semi-civilised tropics which hold their eternal threat to the very existence of mankind! If any doubt the true value of medical endeavour, be it towards true research or towards simple plodding sanitary improvement, and if they still need exhortation to give to organised medicine directly or indirectly whatever help lies in their power, then we urge them to read Dr. Balfour's "War against Tropical Disease."*

*[Balliere, Tindall and Cox, 8 Henrietta Street, Covent Garden, London. Price 12s. 6d. net.]

